# Hospital and Institutional News

**Published:** 1912-06-01

**Authors:** 


### HOSPITAL AND INSTITUTIONAL NEWS.

### OUR HOSPITAL SUNDAY SPECIAL NUMBER.

This year as usual The Hospital, ten days before Hospital Sunday, will distribute among the congregations of the capital a Special Number, dealing exhaustively with the claims of the voluntary hospitals in London to public support, and especially to that of the churches. No effort has been spared to make the Hospital Sunday Special Number this year attractive as well as authoritative reading. The contributors to the Number include the Right Hon. the Lord Mayor of London, Sir Thomas Crosby, M.D., who writes on "The Value of Hospital Sunday." The Rev. H. R. Gamble, rector of Holy Trinity, Sloane Street, has kindly contributed an article on "The Clergy and Hospital Sunday." The Number will also contain "A Layman's Appeal to the Laity," while the technical side of the subject will be treated by the expert hand of Sir Edmund Hay Currie, who, as Secretary of the Sunday Fund from its inception, describes in a special interview the growth and future prospects of the Hospital Sunday movement in London.

### A UNIQUE HOSPITAL AT CAMBRIDGE.

On May 24 the new Research Hospital was opened at Cambridge, and on the previous day the honorary degree of M.A. was conferred by the University upon Mr. R. C. Brown, M.B., F.R.C.P., F.R.C.S., to whose liberality the continuance of the researches undertaken has been very largely due. The new hospital, of which a full account with illustrations appears on pages 223-224, is in the Hills Road, and is fully equipped for the prosecution of research by modern laboratory and clinical methods. In it will be continued the investigation of rheumatoid arthritis and allied diseases which has occupied the Committee ever since the first inception of the idea by Mr. T. S. P. Strangeways and a few other enthusiasts. The disease thus singled out for attack is certainly one of the most mysterious that afflicts mankind; and a solution of its problems would be a real boon to humanity. Notwithstanding the numerous papers which have been published from Cambridge since this research was inaugurated eight or nine years ago, it cannot honestly be said that any brilliant victory over it has yet been achieved. But the work will be so greatly facilitated by this provision of a special building in which to carry it on that more tangible results may well be expected in the future.

### CLINICAL MATERIAL FOR RESEARCH WORKERS.

Mr. R. C. Brown has from the first been one of the keenest supporters of the Cambridge Committee for the Study of Special Diseases, and has founded and endowed a studentship besides providing much of the expensive equipment for the new hospital. The honorary degree conferred upon him is a graceful and well-deserved compliment, and is a sign that the University authorities are fully conscious of the value of the unique institution in their town and of the importance of scientific research. Next to Mr. Brown and Mr. Strangeways must be mentioned Miss Sykes, of Huddersfield, who has contributed very liberally towards this new and novel hospital. Thanks to these and other interested and generous friends, the building has been opened free from debt; but donations and subscriptions for upkeep are still needed. The work of the Committee, it is interesting to know, was originally begun in lodgings; then a villa was rented for the reception of patients, and now a specially constructed hospital has been built. The patients, too, are as unique as the objects of their admission, for rich as well as poor are accepted, and all enter the hospital knowing that the purpose of their stay is to provide clinical material for the research workers.

### THE KING AND PATIENTS' LIBRARIES.

The constant thoughtfulness for hospitals shown by King George has no better example than his recent present of books to the patients' libraries of St. Mary's and St. George's Hospitals. Mr. Carnegie's foundations have produced a weariness in the public mind of all forms of institutional bookshelves, which Lord Rosebery, that expert observer of the fashions and reactions of the hour, did not fail to turn into a capital exercise in the English language not long ago to the delight of those who appreciate style in after-dinner speakers. This skirmish with the subject brought forward the heavier artillery of Mr. Edmund Gosse, and that never-to-be-tracked hare was started: What books should we destroy? The parable of the tares and wheat which were left to fight it out till harvest-time really provides the answer, but our point is that hospital libraries are the only public collection of bookshelves to which the objections against public libraries do not apply. They run no risk, we fear, of overstocking; they are at the mercy of no specialist; they are so far beyond the pale of fashion that they are for the most part thankful for the literary crumbs that fall from the waste-paper baskets of subscribers—magazines, periodicals, and the like. They should not stop there. But what should they include? It is not difficult to answer. When Oscar Wilde left prison he criticised severely the library offered to the prisoners, and suggested, happily enough, that a prison library should contain the Dumas novels. Let that form the basis of an answer, and let the King's example bear fruit, so that those patients who can read may have a chance not limited by stale magazines and works of piety.

### A NEW NAME FOR WOMEN DOCTORS.

A Fellow of the Royal College of Surgeons, who veils his common sense—for we do not believe that he is afraid of being laughed at in that first-rate company of immortal reformers which extends from Gideon to Mr. Jonas Hanway, who was the first bold man to use an umbrella—suggests in the *Times* that lady doctors who possess the degree of M.D. should agree to adopt "some titular prefix expressive of sex." He points out justly that "Matilda Smith, M.D.," is cumbrous, while "Doctor Matilda Smith is as absurd" as Mr. Matilda Smith would be in the case of a lady surgeon. He therefore suggests that lady doctors possessing the M.D. degree should consent to call each other and to be called "doctress." Pedantic objectors he sweeps aside with the revered name of Sir James Murray, the learned philologist, and editor of the new Oxford Dictionary, which gives "doctress," the writer states, as a good and well-established English word, which was "in common use as early as in the sixteenth century." The only other quarter likely to object is that of the lady doctors themselves, and these the writer slily attempts to gain over to his side by remarking that "the frequency of its (doctress) appearance in print would bring into well-deserved prominence the extent to which ladies have captured positions of eminence in the medical profession." That should disarm all opposition, and with all the gravity that this practical reform demands we appeal to the lady doctors for their verdict. It rests with them, with the successors of Dr. Sophia Jex-Blake and other brave assailers of prejudice and reaction. What is the opinion of—Mrs. Scharlieb, M.D.; Mrs. Vaughan-Sawyer, M.D.; Miss Jane Turnbull, M.D.; Miss Davies-Colley, M.D.? What attitude will the Royal Free Hospital's Medical School for Women take up? Let us help the cause of all women doctors by publishing their consensus on this important point.

### THE TEMPORARY CLOSING OF CHICHESTER INFIRMARY.

Ten days ago the amended reconstruction scheme of the Chichester Infirmary, which is to be a memorial to King Edward, began to give work to the builders. Major W. A. Chauncy, indeed, expects that in the course of next week the number of available beds will be interfered with by the progress of the work. The probable number of patients which the semi-closed Infirmary will be able to accommodate is thirty-five. The east sanitary block was abandoned to the builders last Saturday, and on July 1 the eastern half of the main building will also be given up. In about March next year, when the kitchens are in the hands of the contractors, the infirmary will have to close for a short time, and it is hoped to complete the reconstruction in about nine months. To complete "to the best advantage," however, Major Chauncy asks for two things: a balcony for the children's ward, and a nurses' home. Intending benefactors may take courage from the almost finished laundry which Mrs. D. Henty generously gave.

### THE NEW PREMISES OF ULSTER CHILDREN'S HOSPITAL.

The new buildings in Templemore Avenue, Belfast, which the Ulster Hospital for Children and Women undertook a year ago, have been completed, and, after a three days' fête had been held to raise equipment funds, were opened to receive patients. It was in 1882, a hundred and ten years after its foundation, that the gynæcological department was added; the site of the present buildings was obtained in 1891, when they were opened by Mr. J. Blakiston-Houston. In 1905, however, it was seen that extension was imperative, and an appeal for £10,000 was made. Mr. John Stevenson, chairman of the board, Mr. Ernest A. Boas, honorary secretary, and Mr. Robert A. Mitchell, honorary treasurer, worked very hard for this purpose; but the last £1,000 proved exceedingly difficult to raise, until, through the help of Mrs. M'Mordie, £3,000 was subscribed in twelve months. The new buildings consist of four storeys, with blue brick facings below, rough-cast above, and red perforated bricks in the centre. The ground floor is practically given up to the administration, except the north wing which contains a large out-patient department, with its ancillary consulting and ophthalmic rooms Two children's wards occupy the first floor, with fifteen beds in each, and a balcony. Painted tiles, the value of which as a mural decoration in hospitals we discussed recently, representing nursery rhymes, fill the space between the windows. There is also a women's ward for eight beds, a theatre unit, while the nurses' and domestic staff's rooms are on the second and third floors.

### THE MEDICAL SOCIETY AND THE LORD MAYOR.

The Lord Mayor, Sir Thomas Boor Crosby, M.D., F.R.C.S., has been admitted an Honorary Fellow of the Medical Society of London. Advantage was taken of the occasion of the annual oration, which this year was delivered by Mr. S. Bland-Sutton on "Fertilisation in Relation to Pathology," thus to honour the first Lord Mayor in London's history who has ever yet been a medical man. We have already referred to the almost daily proofs which Sir Thomas Crosby gives of the advantages gained by the country and his profession from a medical occupant in this high office; it is not fanciful to believe that these influenced the Society in choosing him to be the recipient of this distinction.

### MR. SYDNEY PHILLIPS ON SAMARITAN FUNDS.

Under the title of "Social Work in Hospitals: the Samaritan Fund," Mr. Sydney Phillips, steward at St. Thomas' Hospital, has published through Longmans, Green, & Co., at the modest price of sixpence, the paper which he read before the Hospital Officers' Association last March on this subject, confining his remarks to London Hospitals. Mr. Phillips states that the first Samaritan Fund was founded at the London Hospital in 1791, at the suggestion of one of its surgeons, Sir William Blizard. Other hospitals followed suit, but with pardonable pride Mr. Phillips claims St. Thomas' to be the real pioneer. The object of a Samaritan Fund he gives in the words of the first report on the subject to be issued by St. Thomas', to provide "some aid subsequent to, and distinct from, the medical and surgical assistance afforded by the hospital." After-care, of course, is the modern term for it. Originally, though not at his hands, "the redeeming of tools from pledge " was a frequent form of assistance, and one that, as the author points out, is still demanded of relief funds in general. The place which after-care has increasingly held in the affections of far-seeing benefactors and testators is then outlined, one of the latest examples being the foundation of the Schiff Home, which has been carefully noted in The Hospital. Mr. Phillips notes also, as did a writer on " The Almoner " in these columns a few weeks ago, the need for co-ordinating after-care with the many other agencies that are allied to it, and also for sifting out those patients which belong properly to the Poor Law. The work of Parochial Relief Committees is then discussed, and the encouragement of self-help by the Samaritan Fund is carefully explained. Mr. Phillips argues, from the standpoint of economy, against hospitals having their own convalescent homes, and compares Samaritan work in London with that of the provinces and in the United States. He concludes by showing that the aim of those who founded the Samaritan Fund has been realised, and that this work " has widened the sphere of usefulness of the hospitals themselves." Mr. Phillips' paper is the illustration of this truth, and the spirit of voluntary work in its happier and more constructive side could have found no more inspiring or authoritative exponent.

### A NEW REVIEW.

The sciences of bacteriology, protozoology, and general parasitology are to have a review all to themselves for the future. This important undertaking is edited by Mr. Foulerton and Dr. Charles Slater, which is sufficient guarantee of a high standard of aim and attainment. The *Review of Bacteriology* is to be published five or six times a year at an inclusive subscription of half a guinea, payable in advance; single copies will not be sold. The first number consists almost exclusively of abstracts of original research from recent publications. These abstracts are well selected from British, American, and foreign sources; are pithy, clear, and to the point; and are one hundred in number. To professional bacteriologists this review is bound to prove a great boon. It is published at 36-38 Whitefriars Street, E.C.

### COAL SAVING AT A MENTAL HOSPITAL.

A considerable saving of coal has been obtained at Cane Hill Mental Hospital by the centralisation of the hot-water system, which was completed rather less than a year ago at a cost of some £3,500. During the ten months ending March 31 last the actual reduction in the consumption of steam coal, making no allowance for a stoppage of the heating system during the last week of March, but for which forty tons more would have been used, as compared with the average consumption for the three preceding years, amounted to 583 tons, which represented a saving of approximately £600. This is the first year since 1893 in which there has been a consumption of less than 3,000 tons. The average consumption during the past ten years has been 3,600 tons annually. The experiment therefore may be regarded as a success, and the example at Cane Hill will perhaps lead other institutions to adopt a centralised hot-water system, where this is not already in force.

### "MEDICAL HOMES FOR PRIVATE PATIENTS."

The 1912 Classified Directory of Medical Homes, with its List of Medical Consultants, which The Scientific Press, 28 Southampton Street, Strand, has now brought out for the seventh year in succession, is an invaluable pocket reference-book for the medical and nursing professions. It was on a leading practitioner's suggestion that a list of private nursing homes, sanatoria, and health resorts should be compiled, and he himself purchased the first copy of a book for which he had long felt a need. The present edition is the most complete which has yet appeared, and notice of any omissions will be gladly accepted by the Editor, who hopes that the list of consultants which has been added will be found useful by country practitioners. All particulars of new institutions should be addressed to the Editor, " Medical Homes," c/o The Scientific Press, Ltd., 28-29 Southampton Street, Strand, W.C. As an ABC of classified homes this modest publication is quite unrivalled. It is the patient's ready-reckoner of where to be ill, and the practitioner's guide as to where to send those of his patients who require institutional treatment, while, in many cases, relying on their own medical man.

### A CROP OF HOSPITAL APPOINTMENTS.

Just at the present moment there is a very unusually large number of visiting staff appointments vacant at the voluntary hospitals in London. Thus the West London Hospital is advertising for a physician to fill the place of Dr. Seymour Taylor, who is time-expired after many years of yeoman service to the hospital. Applications are also invited for the post of assistant physician, which will be filled should one of the present assistant physicians be elected to the senior appointment. The senior assistant physician at the moment is Dr. Harold Pritchard. At Charing Cross Hospital an assistant surgeon is required to fill the gap left by the promotion of Mr. Charles Gibbs in place of the late Sir Frederick Wallis, and also an assistant obstetric physician owing to Dr. T. W. Eden having succeeded Dr. Amand Routh as obstetric physician. Then St. Mark's Hospital for Cancer, Fistula, and other Diseases of the Rectum wants two assistant surgeons; one to fill the gap left by Sir F. Wallis' death, the other apparently as an addition to the present surgical staff. The City of London Hospital for Diseases of the Chest is advertising for a physician to out-patients in succession to Dr. Haldin Davis, who has resigned; there is an honorarium of forty guineas attaching to this post. St. George's Hospital is losing shortly the services of Dr. W. R. Dakin, who retires after many years of notable service as obstetric physician. Dr. A. F. Stabb succeeds to this appointment, and the office of assistant obstetric physician thus left empty is to be filled by the appointment of Dr. G. F. Darwall Smith, who has been obstetric tutor and registrar for some years. At St. Mary's Dr. W. J. Gow has recently been promoted to the rank of obstetric physician, and is succeeded as assistant obstetric physician by Dr. T. G. Stevens. Altogether it is clear that there are plenty of chances for the rising generation in London just now.

### LADY DISPENSERS AND THE INSURANCE ACT.

In order to secure the fullest possible advantages to those employed as dispensers to medical practitioners and public institutions and as chemists' assistants, a Chemists' Friendly Society has been established, with headquarters at 194 St. Vincent Street, Glasgow. Dispensers and chemists' assistants are, on the whole, a healthy class, and it will probably be to their advantage to join a society of their own in preference to a society which is open to all classes. Lady dispensers will be interested to know that there is a women's section of the society.

### POOR-LAW DISPENSERS AND THEIR SALARIES.

Last week we published a paragraph concerning the dissatisfaction with the present scale of salary paid in the Metropolitan area. At a recent meeting of the Lambeth Board of Guardians the question of increasing the salary of the dispenser was considered, and the infirmary committee recommended that Mr. W. Thomas, pharmacist to the infirmary, should have his remuneration increased to £180 per annum, with certain extras valued at £10 per annum. They also considered the question of extra payment in respect of his taking over the dispensing for two additional districts and recommended an extra £20 per annum for this work. Subject to approval by the Local Government Board, the Lambeth Board adopted the recommendations, which were made in view of the high efficiency maintained by Mr. Thomas in the execution of his duties. There is a tendency with the Boards of Guardians to reduce the number of out-district dispensaries and to centralise the work at the infirmary, but, we hope, not to the extent of giving a dispenser more than he can safely superintend.

### A COUNTY DISPENSARY IN WORCESTER INFIRMARY.

A small sub-committee has been appointed to consider the suggestion that three rooms at the Worcester General Infirmary be used by the County Council as a dispensary for tuberculosis cases in connection with the Interim Report of the Departmental Committee on Tuberculosis. The Chairman moved that certain stock be sold and that arrangements be made for an overdraft at the bank of £2,000. Mr. Cuthbert Raymond called the attention of the committee to the fact that by the end of the year it was estimated that £6,078 would be required, and the receipts would only amount to £1,905. That showed, he argued, the necessity for realising those two stocks, which were the only negotiable securities the committee had left, with the exception of £20,000 which stood in the Worcester Corporation Stock. Further expense would be incurred in fitting up the new rooms of the Memorial annexe, in providing the new x-ray apparatus, and in carrying out the Insurance Act when it came into force—an expense which had not yet been estimated. The Chairman agreed that the condition was precarious, but it was impossible to close the infirmary doors. He appealed to fellow-citizens for help. They were doing their best for the city, and he hoped the city would try to do its best for the infirmary. With regard to cases of phthisis in the infirmary, the medical staff do not consider that such cases involve any risk to the other patients, nor do they think it necessary to alter the rules by which cases of incipient phthisis are admissible. It will be very interesting to see what is decided with regard to the use of three rooms as a County Council Dispensary, and the report of the sub-committee will be eagerly awaited.

### THE DATE OF THE METROPOLITAN HOSPITAL DINNER.

We are asked to state that the Hon. Reginald Ailwyn Fellowes will preside at the Annual Festival Dinner, in aid of the Metropolitan Hospital, which will be held at the Whitehall Rooms, Hôtel Métropole, on Monday, July 8, instead of Wednesday, July 10, as previously announced.

### A LADIES TRIUMPH AT CAMBRIDGE.

In the year 1904 the committee of Addenbrooke's Hospital, Cambridge, had to consider the question as to whether the two wards which then existed for children ought not to be closed on account of their cheerless condition. With great reluctance it was decided that it would be prudent to close these wards. Since 1905 all the children have had to be admitted to the main building, the infants into a small ward, and all other children into the various adult wards. This arrangement has been very unsatisfactory, and has only led to many urgent adult cases having been compelled to wait a long time before admission. Soon after the closing of the wards for children a number of ladies banded themselves together under the title of "The Addenbrooke's Hospital Needlework Guild" and determined that they would raise sufficient funds to re-establish new wards for children. They set out to raise not less than £3,000 for this purpose, and have worked diligently and earnestly, making a variety of articles and holding a sale of work each year, with the result that at the end of April 1912 the handsome sum of £2,985 had been realised. On May 23, 1912, the Guild held their annual sale of work for the same object. The sale of work was opened by Lady Albinia Donaldson, wife of the Master of Magdalene College, Cambridge, and supported by the Mayor and Mayoress (Alderman and Mrs. Campkin), Professor J. B. and Mrs. Bradbury, Mrs. Sims Woodhead, Mrs. Ekin, Mrs. Binghey, Mrs. Griffith, president of the Guild, Mrs. Greenwood, Mrs. Stace, Mrs. Stuart, Mrs. Conybeare, Mrs. P. L. Hudson, Mrs. Edwards, Mrs. Flather, Mrs. Cronin, and Colonel T. W. Harding, chairman of finance committee of hospital. The amount realised by the sale was about £120, so that the task which this band of willing helpers set themselves has been accomplished and their zeal and perseverance rewarded. It calls, indeed, for the deepest appreciation, to which undoubtedly the committee of management will give expression.

### A DOCTOR'S DESIGN FOR DONCASTER'S NEW INFIRMARY.

The Royal Infirmary at Doncaster contains sixty-five beds in the wards and seventeen emergency beds for summer use on the balcony. This number has to do duty for the rapidly growing mining town. The coal-pits are now fifteen in number. Recently a medical man published in the local paper a detailed plan of a new hospital on the present site to provide accommodation for 200 patients in the wards and eighty-four on the balconies as well as the necessary quarters for a resident nursing and domestic staff; he also suggested a roof-garden. An architect wrote in approval, adding, however, that the utilisation of the present site would interfere with a proposed new street, which was designed to connect two already existing main thoroughfares. The subscriptions to the infirmary are falling off, and last year's deficit was over £1,000. The question of a new infirmary will not again be allowed to drop now that the general public is slowly awakening to the need for reconstruction.

### THE AFTER-CARE OF DEAF, BLIND, AND CRIPPLED CHILDREN.

Our recent articles under the general heading of "The Defective and Dependent" have certainly formed the most concise summary yet available as to the growth of the after-care principle of recent years. To our list of instances we add that given by Lord George Hamilton, who, as President of the After-care Association for Blind, Deaf, and Cripple Children, 91 Parliament Street, remarks: "The public funds supply the cost of educating these physically defective children, who, though deficient in some physical attributes, are capable, with initial assistance, of earning their own livelihood. When their school life is finished the contributions of the public authorities legally cease, and these poor children have then to fall back upon their own resources and those of their friends. The average cost of starting each child in life is about £10, and it is money well invested." We agree, and all readers of the articles referred to will have appreciated after-care as probably the most inspiriting side of hospital work.

### £30,000 FOR KING'S COLLEGE HOSPITAL.

It is announced that the committee for the removal of King's College Hospital to South London have received from an anonymous donor a cheque for £30,000, being the first instalment of a donation of £50,000 in all to this fund. The donor's only condition apparently is his wish that £2,500 of this amount be specifically allotted to the medical school buildings attached to the new hospital. The whole conditions of the problem before the removal committee are altered by this magnificent donation, and it may now be said that all the means, and, we will add, the responsibilities, which the possession of money gives, are in the hands of and rest upon those responsible for the efficiency of the new institution.

### TRANSPORT STRIKERS AND A LONDON HOSPITAL.

"Owing to the action of the transport strike leaders," wrote Mr. J. G. Broodbank, acting chairman of the Poplar Hospital for Accidents, in East India Dock Road, last Saturday to the lay press, "I think that the public should know that this hospital is being placed in a serious difficulty. The hydraulic power for the lift which conveys patients from the receiving room to the wards has long been supplied to us free by the Port of London Authority. To-day (that is, on Saturday last) the men at the Authority's power station have without notice ceased work and our lift cannot therefore be operated. Every patient, however bad his condition, will now have to be carried up our narrow stairways, and the result must be to give great discomfort in all cases and in some cases to endanger life, to say nothing of the extra expense of engaging special porters, night and day, for the work. When the pressure was cut off three patients and a porter were actually in the lift, which thereupon descended to the basement, and there was the greatest difficulty in getting the patients to their respective wards through the confined approaches to the basement, which is really the coal cellar of the hospital." Fortunately, Mr. Broodbank's connection with the Port enables him to do everything possible to improve matters, but it is feared that a large staff of engineers will be necessary if the lift is to be set at work before the strike ends, and at the moment of writing we regret having to state that the hospital has still to rely upon its narrow stairs, to the risk and discomfiture of many serious cases.

### THE INSURANCE ACT IN YORKSHIRE.

A meeting of Doncaster medical men has been held to consider their position under the Insurance Act, and it is announced that practically all the practitioners in the town, who were present in large numbers, resolved to sign pledges refusing to work under the Insurance Act, and in addition to give up all club practice from a certain date.

### A FEVER HOSPITAL CREATES A RECORD.

Dr. D. L. Anderson, the Medical Officer of Health for Doncaster, reveals in his monthly report that since the outbreak of typhoid fever last January and February the general health has steadily improved, and the fever hospital indeed has been empty for thirty days—which constitutes a record for recent years. There had been no case of infectious disease notified in Doncaster for five weeks. He attributes this recent immunity to the method of following up which is practised under his supervision. Every case that can reasonably be removed to the fever hospital is so treated, and the rest are rigidly isolated. A special register is also kept, and each disease is recognised in the entries by a distinctive coloured ink. Other routine details include such items as the date of the visit and subsequent revisits of the sanitary inspector, milk and bread supply, and so forth. The number of contacts are also followed up as in smallpox, only the system is extended to each notifiable disease.

### THE EVOLUTION OF A FEVER HOSPITAL.

THE application of the Dewsbury District Joint Hospital Board for leave to borrow £2,000 to erect a separate building containing fourteen beds at the Infectious Diseases Hospital at Chickenley Wood was not opposed at the Local Government Board inquiry. Dr. Hammerton, medical superintendent, said that additional provision for diphtheria patients was urgently needed, and Mr. H. Ellis, the town clerk of Dewsbury, remarked that the hospital served a population of 65,000. It was opened in 1905 to accommodate fifty-two patients. At first, only scarlet fever and typhoid cases were admitted, but the applications for admission from diphtheritic patients have made it necessary to devote a portion of the typhoid ward to their use. Last year thirty-seven were admitted. This illustrates another side of the problem of avoiding cross infection in provincial hospitals to that which we drew attention to the other day.

### THE REVIVAL OF HOSPITAL SUNDAY IN SHEFFIELD.

A JOINT meeting of Sheffield clergy, ministers, and representatives of other religious bodies has been held in Sheffield, a sub-committee of which has adopted a new scheme for organising the fund. This scheme, which is not intended to interfere in any way with the existing Hospital Sunday Committee, was adumbrated in a capital speech by the Rev. S. T. G. Smith, who presided. He pointed out that the clergy were really ashamed of the comparatively small amount that is given by the various churches. The clergy, however, Mr. Smith proceeded, do not take upon themselves the blame, for they have had so very little to do with organising the fund in Sheffield that it is not really their fault that things have fallen into their recent lamentable condition. The new proposal will be a mutual and combined one between the clergy and the hospitals, and the general committee which has been appointed will collect information, provide literature, arrange details of the annual collections, and possibly organise further meetings. Proposed by Dr. G. S. Swan, vicar of Pitsmoor, seconded by Mr. J. H. Freeborough, and endorsed by the chairman of the Sheffield Royal Hospital, Mr. Philip K. Wake, the scheme was carried. Our recent criticisms provoked the usual disclaimers, but it is satisfactory to note that the clergy in Sheffield have subsequently undertaken their reforms themselves. For that they deserve every credit, and we shall hope to chronicle when the time comes the success of this attempt at reviving Hospital Sunday.

### ANGLOPHOBIA AND ENGLISH DOCTORS.

THE reception at Potsdam of the representatives of the Royal Institute of Public Health, who will arrive in Berlin on July 25, is, according to the Berlin correspondent of the *Times*, causing local heartburnings of a peculiarly unfortunate kind. At a meeting last week, he writes, of the Potsdam Town Council a proposal by the Mayor and Aldermen to grant the sum of £45 towards the expenses of entertainment was defeated after a heated debate by a large majority. Except to an Anglophobe journal, he adds, the incident has occasioned here some resentment and surprise. Hospitality is a virtue that may well be expected of a municipality, which should be the first to recognise the claims of public health officials to common courtesy on their health mission.

### THE OBJECT AND VALUE OF EGG COLLECTIONS.

IT does not always occur to those who are interested in our voluntary hospitals that there are many ways other than contributing in money by which they can render valuable assistance. One of the least hackneyed of these ways is by contributing gifts in kind, which not only afford a welcome change in the daily dietary but materially reduce expenditure. There are many whose slender income precludes them from subscribing to their local hospital. Now a gift in kind does not confine its possibilities to adults, for the children can take their share in and enjoy this work. In Cambridge the experience is that the children are ever ready to help Addenbrooke's Hospital, as the following instances show: The girls in St. Luke's Schools contributed sufficient to provide two comfortable chairs for the children's ward, thereby supplying a long-felt need. Last year an appeal made to the various schools in the district brought in about 10,000 new-laid eggs. In March this year the headmasters, headmistresses, and teachers from 150 schools were invited to attend a special meeting held at Addenbrooke's Hospital to consider the question as to what steps could be taken to organise egg collections in schools on a more systematic basis. The meeting was well attended, being presided over by the Headmaster of Duxford School, and the result up to the present has been that the children have contributed something like 15,000 new-laid eggs.

### THE STRIKE'S EFFECT ON THE DRUG MARKET.

BUSINESS in the Drug market has been quiet during the week owing to the intervention of the Whitsuntide holiday and the transport strike. Considerable inconvenience has already been caused by the strike owing to the difficulty of landing and delivering drugs, but as yet no important advances in price can be directly traced to this cause. Of changes in the value of drugs there have been few during the week. Buchu leaves are again dearer, and the tendency is towards still higher prices. Cocaine is tending lower, but as yet makers have not announced a reduction in their quotations. Manufacturers of codeine have announced a further reduction in the price of this alkaloid following on the lower tendency in opium values. Morphine is also tending lower.

### TO OUR READERS.

CONTRIBUTIONS are specially invited from any of our readers to these columns. They should deal with topical subjects and news. They must be authenticated for the information of the Editor only. The minimum payment if published is 5s. There is no hard-and-fast rule as to space, but notes of about twenty lines in length are preferred.

